# A System-Based Review on Effects of Glucagon-Like Peptide-1 Receptor Agonists: Benefits vs Risks

**DOI:** 10.7759/cureus.78575

**Published:** 2025-02-05

**Authors:** Lynn P Fadel, Gigi Thao, Tanvi Chitre, Edwin D Rojas, Maria Nguyen Fricko, Valerie Domingo, Brigita Budginas, Lorenz Carmelo Guerrero, Maria Ghatas, Niloufar T Arani, Niki Tabatabai, Sudhakar Pemminati

**Affiliations:** 1 Department of Biomedical Education, California Health Sciences University College of Osteopathic Medicine, Clovis, USA; 2 Department of Biomedical Education, Noorda College of Osteopathic Medicine, Provo, USA

**Keywords:** endocrinology and diabetes, glucagon-like peptide-1 receptor agonists, polycystic ovarian disease, types 2 diabetes, weight loss and obesity

## Abstract

Glucagon-like peptide 1 (GLP-1), an incretin hormone primarily secreted by L-cells in the gut, prompts insulin release, thus reducing blood sugar levels and causing weight loss by inducing feelings of fullness while curbing appetite. GLP-1 receptor agonists (GLP-1 RAs) mimic its effects, proving highly effective in managing type 2 diabetes mellitus (T2DM) and facilitating weight loss. While predominantly approved for T2DM and obesity, GLP-1 RAs also hold promise for treating other conditions like heart and kidney disease, with ongoing research exploring additional therapeutic applications. These agonists exhibit diverse effects within different organ systems, influencing conditions such as psoriasis, polycystic ovarian syndrome (PCOS), thyroid disorders, neurodegenerative diseases, and cardiopulmonary dysfunction. This study aims to comprehensively review the impact of GLP-1 RAs on various body systems, emphasizing both positive and negative effects while addressing existing knowledge gaps in the literature. By enhancing understanding of the diverse effects of GLP-1 RAs, this study aims to contribute to a broader awareness of their therapeutic potential. This systemic review uses Preferred Reporting Items for Systematic Reviews and Meta-Analyses (PRISMA) guidelines to ensure a robust and transparent search process, aiming to minimize bias and maximize the retrieval of pertinent studies for review, where past research on GLP-1 RAs’ interactions with various bodily systems were analyzed. The hypothesis posits that a systems-based review of GLP-1 RA mechanisms will reveal positive and negative effects across multiple organ systems, providing comprehensive insights into the hormone's physiological impact. This systematic review will assess the appropriateness of GLP-1 RAs in various aforementioned patient health states, shedding light on their potential impacts on comorbidities. With the surge in popularity of GLP-1 RAs for weight loss and diabetes management, this study aims to enhance patient understanding and informed decision-making regarding these medications, countering trends driven by celebrity endorsements and promoting better healthcare outcomes.

## Introduction and background

Incretins are a group of metabolic hormones that stimulate a decrease in blood glucose levels. They are released after eating and amplify the secretion of insulin from the pancreas. The two main incretins are gastric inhibitory polypeptide (GIP) and Glucagon-like peptide-1 (GLP-1). This systematic review will focus on GLP-1, which is secreted from the L-cells in the distal ileum and colon within minutes of oral glucose ingestion [1]. In β-cells, binding of GLP-1 to its receptor leads to activation of adenylate cyclase (AC) and subsequently to an increase in cyclic adenosine monophosphate (cAMP) stimulate insulin secretion from β-cells of the pancreas and exerts further glucoregulatory actions such as slowing gastric emptying, inhibiting glucagon secretion, and promoting satiety. GLP-1 receptors can be found in tissues other than the pancreas, such as in the central nervous system, gastrointestinal tract, stomach, skin, reproductive system, and cardiovascular system [1].

In individuals with conditions like T2DM, the incretin effect is diminished, leading to decreased secretion of GLP-1 by the L-cells. Though the reason why this occurs is still being investigated, GLP-1 RAs have been the current leading therapeutic option. The injectable treatment is deemed more effective than the therapeutic administration of native GLP-1 or inhibition of enzymatic degradation by dipeptidyl peptidase IV (DPP-4) [2].

The incretin effect refers to the phenomenon where oral glucose ingestion leads to a greater increase in insulin secretion than intravenous administration of the same amount of glucose, mediated by GLP-1 and GIP. Following a meal or oral glucose load, GLP1-RAs use these mechanisms to reduce blood glucose: enhance glucose-dependent insulin release from pancreatic β-cells, decrease endogenous glucose production, reduce food intake, increase energy expenditure, and delay gastric emptying [3]. GLP-1 RA can also decrease pancreatic β-cell apoptosis while promoting their proliferation.

In the cardiovascular system, GLP-1 RAs can improve left ventricular ejection fraction, myocardial contractility, coronary blood flow, cardiac output, and endothelial function while reducing infarction size and overall risks for a cardiovascular event [2]. Other functions of GLP-1 include increased glucose uptake in the muscles, decreased glucose production in the liver, neuroprotection, and increased satiety due to direct actions on the hypothalamus [2].

**Figure 1:**
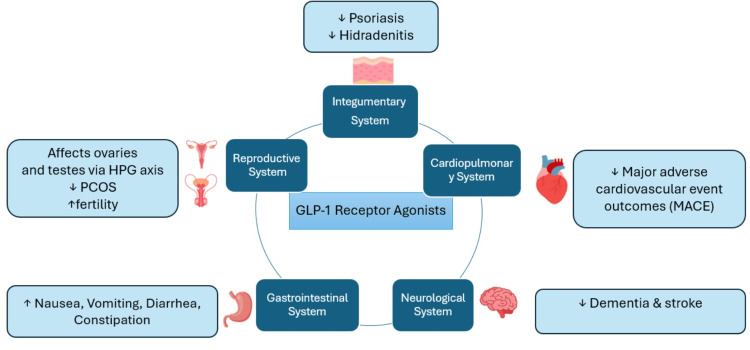
The effect of glucagon-like peptide-1 receptor agonists (GLP-1RA) on various body systems Image credits: Maria Nguyen Fricko HPG: hypothalamus-pituitary-gonadal; PCOS: polycystic ovarian syndrome

GLP-1 RAs are typically used as an adjunct to diet and exercise to improve glycemic control in adults with T2DM. They are also advertised as a treatment for overweight and obese individuals. The current guidelines are to first attempt to improve the condition with a proper diet and adequate exercise and then use the drug as a supplement if the desired goal of care is not met [4]. This literature review aims to provide detailed multisystemic effects that GLP-1 RAs have beyond both the endocrine and gastrointestinal systems as illustrated in Figure 1. Having a well-rounded, systemic understanding of the risks and benefits of this polarizing drug class will potentially uncover how it may impact and alleviate different disease processes beyond diabetes and obesity.

## Review

Methods

Search Strategy

The search strategy was developed using the California Health Sciences University (CHSU) library and included the following databases: PubMed, Web of Science, SCOPUS, and Embase. The search was limited to studies published from 2005 to 2024. Specific keywords and Medical Subject Headings (MeSH) terms were employed for each thematic area to ensure a thorough and targeted search. The Preferred Reporting Items for Systematic Reviews and Meta-Analyses (PRISMA) guidelines were followed for an efficient search process, aiming to minimize bias and maximize the retrieval of pertinent studies for review [5].

For the integumentary system, the terms used included “GLP-1 receptor agonists” AND “skin” as well as “GLP-1 receptor agonists” AND “dermatology.” For endocrinological aspects, the terms “GLP 1 receptor agonists” AND “thyroid cancer”, “GLP-1 receptor” AND “Endocrine pancreas”, “GLP-1” AND “adrenal glands”, and “GLP-1” AND “medullary thyroid cancer” were employed. For reproductive health, search terms were “GLP-1 receptor agonists” AND “female reproduction” and “GLP-1 receptor agonists” AND “male reproductive health.” The neurological focus utilized terms such as “Anti-inflammatory” AND “glucagon-like peptide 1,” “Diabetes Mellitus, Type 2/drug therapy”[Mesh] AND "Blood Glucose"[Mesh] AND "Obesity"[Mesh], and a combined query using ("tirzepatide"[Supplementary Concept] OR "tirzepatide"[All Fields]) AND ("inflammation"[MeSH Terms] OR "inflammation"[All Fields]) AND ("nervous system"[MeSH Terms] OR ("nervous"[All Fields] AND "system"[All Fields]) OR "nervous system"[All Fields])). Additional neurological search terms included (“Glucagon-Like Peptides"[Mesh]) AND "Animals"[Mesh]) AND "Cognition"[Mesh], “neurological” AND “incretin,” “GLP psychiatric,” “semaglutide adverse effects,” and “GLP-1 neurological.” In the gastrointestinal category, searches included “GLP-1 side effects,” “GLP-1” AND “Gastrointestinal,” “Semaglutide side effects,” “GLP-1 side effects” AND “Fibrosis,” “GLP-1 receptor agonists” AND “Pancreas,” “GLP-1 receptor agonists” AND “gallbladder,” and “GLP-1 receptor agonists” AND “semaglutide safety.” For cardiovascular research, terms included “GLP1 agonists” AND “cardiovascular.”

Inclusion & Exclusion Criteria

The populations that were examined were GLP-1 RA users, regardless of their T2DM diagnosis. All age groups, races/ethnicities, and genders were considered. The following study designs were included: literature reviews, systematic reviews using PRISMA, case reports, case-control studies, clinical trials (i.e., double-blinded, randomized, and placebo-controlled trials), and longitudinal studies. Articles that were excluded from the search were either written in a different language, included pediatric populations, had incomplete data, or did not have adequate statistical analysis.

Study Selection

**Figure 2:**
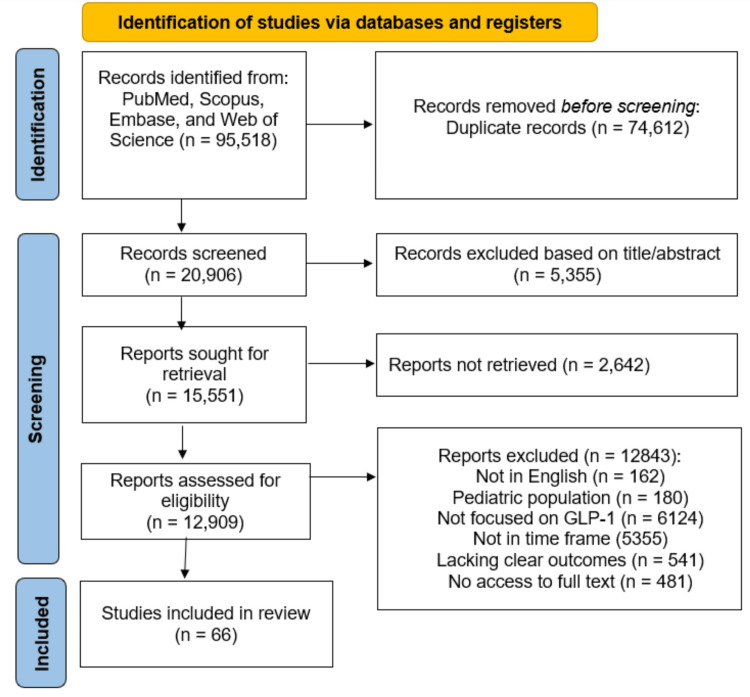
Study of selection for systematic review using PRISMA guidelines PRISMA: Preferred Reporting Items for Systematic Reviews and Meta-Analyses

Two independent reviewers screened titles and abstracts for eligibility. Full-text articles of potentially relevant studies were then assessed against the inclusion and exclusion criteria. Discrepancies between reviewers were resolved through discussion, and the advice of the principal investigator was considered (Figure 2).

Effects of GLP-1 RAs on the integumentary system

Throughout the literature review, numerous studies have explored the effects of GLP-1 RA on psoriasis in patients with T2DM focusing on distinct immune mechanisms and clinical outcomes. Hogan et al. conducted a case-series study aimed to investigate whether the improvements in psoriasis severity seen with GLP-1 analog therapy, specifically liraglutide, were linked to direct effects on invariant natural killer T (iNKT) cells. They measured the numbers of circulating and psoriatic plaque iNKT cells before and after treatment. The results showed that the Psoriasis Area and Severity Index (PASI) improved significantly in two patients. Participant 1’s PASI decreased from 13.2 to 10.8, and participant 2’s from 4.8 to 3.8 over six weeks. These findings suggest that liraglutide could modulate iNKT cells, reducing psoriasis severity [6]. In contrast, Buysschaert et al. conducted a case-series study of 17 patients to assess the effects of liraglutide and exenatide on both the clinical and histopathological aspects of psoriasis. Their study evaluated changes in PASI scores and focused on immune markers such as γδ T cells and interleukin (IL)-17 expression in psoriatic lesions. After 16-20 weeks of treatment, the mean PASI decreased from 12.0 ± 5.9 to 9.2 ± 6.4 (P = 0.04), and histological analysis revealed reduced epidermal thickness in the treated patients. Additionally, the percentage of γδ T cells in psoriatic lesions decreased from 6.7 ± 4.5% to 2.7 ± 3.8% (P = 0.05) in those who showed improvement or stabilization of PASI, with a corresponding reduction in IL-17 expression. This study highlighted the role of γδ T cells and IL-17 in psoriasis and demonstrated that GLP-1 agonists could exert anti-inflammatory effects through these pathways [7].

Similarly, a study conducted by Xu et al., which focuses more on the direct impact of liraglutide on PASI and Dermatology Quality of Life Index (DQLI) over 12 weeks, had similar trends. Among seven patients, there was a mean PASI decreased from 15.7 (1.5-31.3) to 2.0 (0.3-8.7) (P = 0.03); the mean DQLI decreased from 22 (8-27) to 4 (0-10) (P = 0.001), meaning that there is a positive change in the patient’s quality of life. These findings indicate that GLP-1 RA may effectively reduce psoriasis severity in patients with T2DM by influencing immune cell activity, inflammatory markers, and quality of life. Another common concern regarding T2DM is foot ulcers [8]. A study by Nagae et al. investigated the effects of the GLP-1RA, liraglutide, on wound healing, particularly in keratinocyte migration, and its role in treating difficult-to-heal diabetic foot ulcers. Liraglutide was found to induce keratinocyte migration by activating the PI3K/Akt signaling pathway, as demonstrated in vitro using HaCaT cells and confirmed in vivo through enhanced wound healing in mice. These findings suggest that via GLP-1RA activation, liraglutide promotes keratinocyte migration in the six mice and may offer a therapeutic approach for improving diabetic wound closure. Results were statistically significant, p < 0.05 [9].

With the rise in popularity of GLP-1 RAs, it’s common to overshadow the side effects due to the overwhelming benefits. In a PRISMA analysis conducted by Salazar et al., there is a pool of 33 patients who experienced several unique dermatological reactions to their treatment. The most common findings were dermal hypersensitivity reactions (33.3%) and eosinophilic panniculitis (30.3%). Other reactions included bullous pemphigoid in 9.1% of cases, morbilliform drug eruptions in 6.1%, and angioedema in 6.1%, along with seven isolated cases of various other skin reactions. Exenatide ER was linked to 57.6% of the reactions (19 cases), followed by liraglutide (six cases), dulaglutide (five cases), and subcutaneous semaglutide (three cases). Of the 33 patients, 30 fully recovered, experiencing either no or mild post-inflammatory changes, although three reports did not mention the disease progression. In response, 31 patients permanently stopped the offending medication, while two underwent successful desensitization [10]. To further demonstrate dulaglutide’s propensity to cause dermatological reactions, a case study by Bianchi et al. analyzed the case study of a 45-year-old woman with T2DM who started weekly dulaglutide due to metformin failure. After her fourth dose, she developed an itchy injection site reaction, and after the sixth dose, a widespread delayed urticaria-like rash. The rash resolved after stopping dulaglutide and taking cetirizine. Three months later, skin tests showed a delayed positive reaction to intradermal dulaglutide at higher concentrations [11]. Despite these medications' overall therapeutic benefits, the variety of dermatological reactions associated with GLP-1 RAs underscores the importance of vigilance in monitoring patients for adverse effects.

Another concern regarding GLP-1 RA use that has arisen in recent years due to a clinical trial with liraglutide was an increased risk of melanoma; however, further investigation in a population-based cohort study has shown no association of increased risk of melanoma or non-melanoma skin cancer with GLP-1 RAs use compared to sulfonylureas in patients with T2DM [12]. Despite these promising results, more research needs to be conducted to account for the lack of generalizability to the diabetes population and that there could be variability in the reporting of skin cancer as this study was conducted using a clinical database in the United Kingdom that includes over 2000 primary care practices.

There is evidence that GLP-1 RAs can have an anti-inflammatory effect in those suffering from psoriasis via decreased pro-inflammatory cytokine production promoted by TNF-α [13]. Obesity and psoriasis have been shown to correlate, presumably due to obesity causing an inflammatory state that can worsen autoimmune conditions such as psoriasis [14]. Biopsies of psoriatic lesions demonstrate GLP-1 receptor up-regulation due to immune cell infiltration [15, 16]. Thus, treatment of obesity and T2DM with GLP-1 RAs can help improve the severity of symptoms and psoriatic lesions. Studies of GLP-1 RAs’ effect on individuals with both obesity and psoriasis have exhibited reduced psoriasis areas and psoriatic severity along with improved glucose tolerance [14]. While weight loss induced by GLP-1 RAs can have a positive impact on psoriasis symptoms, GLP-1 RAs seem to have its own independent effect on psoriasis and chronic inflammation through its anti-inflammatory properties and cytokine suppression [17].

In conclusion, GLP-1 RAs demonstrate substantial potential in managing conditions like psoriasis and diabetic foot ulcers, owing to their anti-inflammatory effects, cytokine suppression, and glucose regulation. While weight loss contributes to these benefits, GLP-1 RAs exert independent therapeutic effects on inflammation and skin healing. However, the risk of dermatological side effects warrants careful patient monitoring to balance benefits and risks. The findings are summarized in Table 1.

**Table 1:** Benefits and risks on the integumentary system B: Benefits; R: Risks; GLP-1 RA: Glucagon-like peptide-1 receptor agonist; iNKT cells: invariant natural killer T cells; PASI: Psoriasis Area and Severity Index; T2DM: Type 2 Diabetes Mellitus; DQLI: Dermatology Quality of Life Index; HbA1c: Hemoglobin A1c; BMI: Body Mass Index; IL-17: Interlukin-17.

Author & year	Study population	Country	Benefits/Risks
Liraglutide and exenatide: Buysschaert et al., 2014 [7]	N = 17	Belgium	B: In 16-20 weeks, the mean PASI decreased from 12.0 ± 5.9 to 9.2 ± 6.4 (P = 0.04). Reduced epidermal thickness in the treated patients. The percentage of γδ T cells in psoriatic lesions decreased from 6.7 ± 4.5% to 2.7 ± 3.8% (P = 0.05) with a corresponding reduction in IL-17 expression.
Liraglutide: Nagae et al., 2018 [9]	N = 6	Japan	B: Promotes keratinocyte migration in the six mice and may offer a therapeutic approach for improving diabetic wound closure (p < 0.05)
Exenatide ER, liraglutide, dulaglutide, and semaglutide: Salazar et al., 2024 [10]	N = 33	Germany	R: Dermal hypersensitivity reactions (33.3%), eosinophilic panniculitis (30.3%). bullous pemphigoid (9.1%), morbilliform drug eruptions (6.1%), and angioedema (6.1%).
Liraglutide: Pradhan et al., 2024 [12]	N = 439,867	United Kingdom	B: GLP-1 RAs were not associated with an increased risk of melanoma or non-melanoma skin cancer compared to sulfonylureas in patients with T2DM.
Liraglutide: Ramis et al., 2023 [14]	N = 20	Spain	B: There was an improvement in weight loss and DLQI. However, weight loss from the liraglutide did not have any correlation to inflammatory parameters related to psoriasis or PASI. Anti-inflammatory effects. Liraglutide treatment was found to be effective and safe for patients that had both obesity and psoriasis.

Effects of GLP-1 RAs on the endocrine system

The endocrine effects of GLP-1 RAs have been extensively studied across multiple domains, including thyroid function, the endocrine pancreas, and renal systems. Each aspect has revealed a mix of therapeutic promises and potential risks.

Bezin et al.’s study using the French National Health Database highlighted significant associations between GLP-1 RA use and thyroid cancer risk in the domain of thyroid function. In this nested case-control analysis of 3,746,672 patients with T2DM, 2,562 cases of thyroid cancer were identified and matched with 45,184 controls. The study found an increased hazard ratio (HR) of 1.46 (95% CI: 1.23-1.74) for current GLP-1 RA users compared to non-users. Moreover, cumulative use of 1-3 years was associated with an HR of 1.58 (95% CI: 1.27-1.95) and use beyond three years showed an HR of 1.36 (95% CI: 1.05-1.74) [18]. Similarly, Silverii et al.’s meta-analysis of 64 randomized controlled trials (RCTs) involving 46,228 GLP-1 RA users found an overall increased risk of thyroid cancer with a Mantel-Haenszel odds ratio (MH-OR) of 1.52 (95% CI: 1.01-2.29; p = 0.04). However, no significant associations were found for specific subtypes like papillary or medullary thyroid cancer [19]. In a Scandinavian cohort study from three countries, GLP-1RA use was not associated with a substantially increased risk of thyroid cancer over a mean follow-up of 3.9 years [20]. These findings suggest a nuanced risk profile, emphasizing the need for monitoring, particularly with prolonged GLP-1 RA use.

The effects on the pancreas have been a focal point for investigating the therapeutic benefits and potential malignancy risks of GLP-1 RAs. Ayoub et al. analyzed a cohort of 7,146,016 individuals with T2DM and identified 721,110 GLP-1 RA users after propensity score matching. Over a seven-year follow-up, the study found a significantly reduced pancreatic cancer incidence among GLP-1 RA users (0.14%) compared to non-users (0.20%), corresponding to a 31% reduced risk (risk ratio [RR]: 0.69; 95% CI: 0.639-0.752; p < 0.0001) [21]. Similarly, Dankner et al., in their population-based cohort study of 543,595 patients, reported no increased risk of pancreatic cancer associated with GLP-1 RA use compared to basal insulin users. Adjusted HRs for pancreatic cancer risk decreased over time, with an HR of 0.22 (95% CI: 0.11-0.41) in the first year and 0.32 (95% CI: 0.13-0.76) during years two through four [22]. These findings highlight the potential protective effects of GLP-1 RAs in the pancreatic context, though long-term data remain critical for definitive conclusions.

Renal endocrine effects of GLP-1 RAs have also been explored, with studies revealing potential benefits in nephropathy and sodium regulation. Zhang et al. conducted a randomized 16-week trial involving 31 patients with T2DM and microalbuminuria, comparing exenatide to glimepiride. Exenatide significantly reduced urinary albumin excretion, urinary transforming growth factor beta-1 (TGF-β1) levels (p < 0.01), and type IV collagen (25.3% reduction, p < 0.005) compared to minimal changes in the glimepiride group [23]. These findings suggest GLP-1 RAs’ capacity to mitigate diabetic nephropathy progression. Similarly, Mendis et al. demonstrated that a single dose of exenatide increased the urinary sodium/creatinine ratio significantly within two hours (p < 0.05), indicating natriuretic effects [24]. However, Linnebjerg et al. highlighted challenges in elderly patients between the ages of 45 to 65 and greater than 75 with impaired renal function which showed that exenatide dose should be adjusted based on renal function and age in elderly type 2 diabetic patients [25].

The interplay between GLP-1 RAs and endocrine axes was notably investigated by Gil-Lozano et al., who examined prolonged exendin-4 administration in rodent models. The study observed disrupted circadian rhythms of corticosterone secretion, with levels significantly elevated during trough hours and reduced during peak hours after nine days of treatment (p < 0.01). Aldosterone levels and adrenal weights also increased significantly, suggesting adrenal hypertrophy and heightened hypothalamus-pituitary-adrenal (HPA) axis activity [26]. These findings highlight potential endocrine side effects that may not fully translate to human bodies but warrant further exploration in clinical contexts.

While the thyroid and pancreas findings suggest relatively consistent trends across human studies, renal effects exhibit more significant variability depending on renal function and study design. For example, while Zhang et al. and Mendis et al. highlighted nephroprotective and natriuretic benefits, Linnebjerg et al. highlighted potential risks in patients with advanced renal impairment in elderly patients. These differing findings necessitate tailored clinical approaches based on individual patient profiles, such as age and pre-existing conditions, particularly those with compromised renal function.

GLP-1 RAs demonstrate complex endocrine interactions that balance therapeutic efficacy with potential risks. The apparent protective effects on the endocrine pancreas and nephropathy contrast with thyroid cancer risks, particularly with prolonged use. These findings emphasize the importance of individualized risk-benefit assessments and the need for further long-term studies to refine our understanding of GLP-1 RAs’ endocrine impacts across diverse patient populations. The findings are summarized in Table 2.

**Table 2:** Benefits and risks on the endocrine system B: Benefits; R: Risks; GLP-1 RA: Glucagon-like peptide-1 receptor agonist; T2DM: Type 2 Diabetes Mellitus; HR: Hazard ratio; MH-OR: Mantel-Haenszel odds ratio; TGF-B1: Transforming growth factor beta-1; ESRD: End-stage renal disease; HPA axis: Hypothalamus-pituitary-adrenal axis

Author & year	Study population	Country	Benefits/Risks
Liraglutide Semaglutide Exenatide: Bezin et al., 2023 [18]	N = 2,562	France	B: Improved glycemic control (lowers blood glucose levels aiding in T2DM patients, weight loss, reduction of cardiovascular risk factors, decreased major cardiovascular events. R: Thyroid C-cell tumors in rodent studies but in human studies such has been shown between GLP-1 agonists and thyroid cancer.
Liraglutide, semaglutide, exenatide, dulaglutide, lixisenatide: Silverii et al., 2023 [19]	N = 40,000	Italy	B: Improved glycemic control, weight loss, and renal protection against diabetic kidney disease progression. R: Human data did not show a significant enough increase in the risk of thyroid cancer in humans. Animal studies have confirmed a link, but human studies have not.
Exenatide, liraglutide, dulaglutide: Ayoub et al., 2024 [21]	N = 7,146,016	United States of America	B: There was a 31% decrease in pancreatic cancer.
Dankner et al., 2024 [22]	N = 543,595	Israel	B: There was no excess risk associated with pancreatic cancer in patients with T2DM.
Exenatide: Zhang et al., 2012 [23]	N = 31	China	B: There was significantly reduced urinary albumin excretion, urinary TGF-β1 levels, and type IV collagen, suggesting a reduction in the progression of diabetic nephropathy.
Exenatide: Mendis et al., 2012 [24]	N = 8	United Kingdom	R: A single dose of exenatide increased the urinary sodium/creatinine ratio significantly within two hours.
Exenatide: Linnebjerg et al., 2011 [25]	N = 31	Denmark	R: ESRD patients had reduced clearance of exenatide, clearance was substantially reduced from 3.4 L/h in normal renal function to 0.9 L/h.
Exendin-4: Gil-Lozano et al., 2013 [26]	N = 6-15 per group	Canada	R: Disruption of circadian rhythms of corticosterone secretion, with levels significantly elevated during trough hours and reduced during peak hours after nine days of treatment (p < 0.01). Aldosterone levels and adrenal weights also increased significantly.

Effects of GLP-1 RAs on the reproductive system

The role of GLP-1 RAs on the reproductive system is largely rooted in their weight loss propensities. The hypothalamus-pituitary-gonadal (HPG) axis is an important regulator of reproduction in individuals both assigned female at birth (AFAB) and assigned male at birth (AMAB). The mechanism involves a GnRH production that increases LH or FSH release from the pituitary gland to stimulate the production of androgens in the testis or ovaries. This usually includes a G-alpha S mechanism that causes a phosphorylation cascade to regulate various downstream genes. Due to insulin's expansive role in various systems, it is unsurprising that there are some effects on the HPG axis as well. For example, increased plasma concentrations of insulin can affect the pulsatile release of GnRH leading to an enhanced androgen synthesis as well as directly stimulating receptors on theca cells [27].

**Figure 3:**
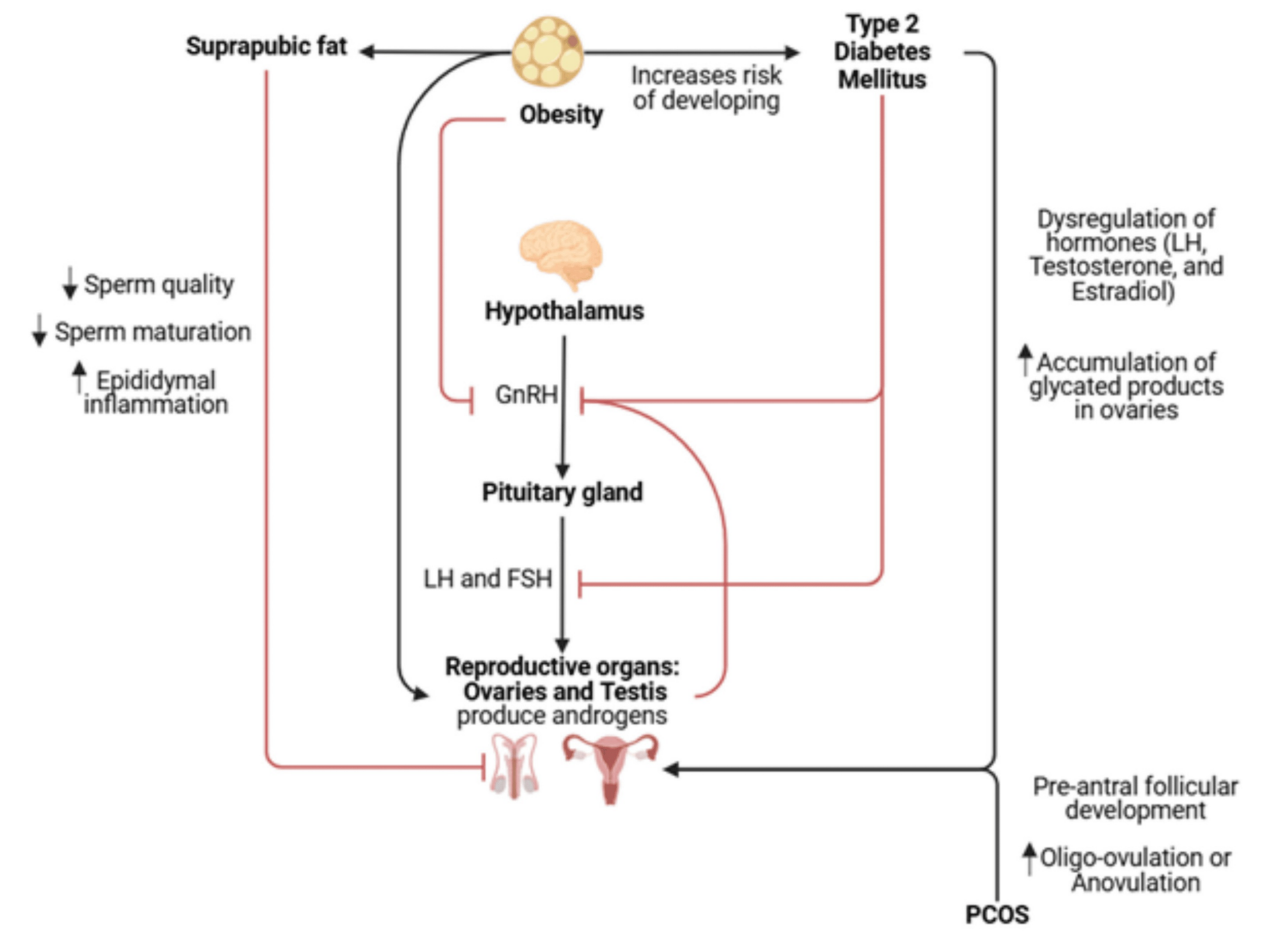
Role of obesity and insulin resistance on ovaries and testis leading to variable feedback on HPG axis. GnRH: Gonadotropin-releasing hormone; LH: Luteinizing hormone; FSH: Follicle stimulating hormone; PCOS: Polycystic ovary syndrome Image credit: Gigi Thao

Obesity can cause secondary hypogonadism, which leads to reproductive abnormalities via metabolic issues like insulin sensitivity [26]. For this reason, investigating weight-loss strategies and metabolic issues that stem from chronic issues like T2DM may give insight into treating complex reproductive issues like PCOS, and female and male infertility. The primary measures of infertility are “anovulation, abnormal hormone levels, decreased pregnancy rates, and longer times to achieve pregnancy” [28]. The use of GLP-1 RAs, which enhance insulin release while suppressing appetite, is worth exploring to better understand its potential effects in both the female and male reproductive systems (Figure 3).

Effects of GLP-1 RAs on Fertility in Females with PCOS

The focus on PCOS is important because of its prevalence of 5-10% among individuals AFAB of reproductive age. It is characterized by oligomenorrhea, hyperandrogenism, and infertility [29]. Current treatment includes a mixture of lifestyle and pharmacological changes, which are important in preventing future complications like atherosclerosis [30]. There is a close relationship between insulin resistance and PCOS, where over 50% of women with PCOS are also overweight, obese, or have insulin resistance [29]. As obesity is currently the mainstay of fertility treatment specifically in the context of PCOS, treatment with GLP-1 RAs holds a lot of promise [31].

Although there are multiple underlying mechanisms for PCOS, it is highly suspected that metabolic dysfunction plays some role in clinical pathology. Two major opposing metabolic signals exist that can occur at the same time and further alter the female reproductive system (Figure 3). Firstly, altered adipose tissue metabolism can increase insulin resistance and modify the pulsatile release of GnRH from the hypothalamus. This increases the LH and FSH ratio leading to a loss of the pre-ovulatory LH surge required to produce the estrogen necessary for follicular maturation. The lack of LH will signal the HPG axis to continue producing GnRH in hopes of increasing downstream products [29]. Eventually, this leads to the development of oligo-anovulation, polycystic ovaries, and hyperandrogenism categorized under PCOS. In another mechanism, obesity increases the production of androgens in the ovaries, and these are converted to estrogen which downregulates the production of GnRH, and thus LH and FSH production [31]. This imbalance of hormone levels leads to dysfunctional menstrual cycles and eventual infertility.

Therefore, treating metabolic disorders such as diabetes mellitus and obesity may show an improvement in PCOS symptoms. Since GLP-1 has a regulatory role for insulin while decreasing appetite, introducing GLP-1RA can increase insulin sensitivity, allowing for some recovery of the LH and FSH ratio [29]. The visceral fat deposition seen in obesity and thus PCOS can increase insulin resistance and hyperinsulinemia, which further exacerbates the problem [31]. Normalization of LH and FSH levels can encourage follicular maturation and improve some symptoms of PCOS [29]. Weight reduction using GLP-1 RAs is a viable method to improve PCOS symptoms and also increase fertility. A retrospective cohort study cited by Cena et al. [31] demonstrated that overweight or obese women with infertility who achieved 10% weight loss had “significantly higher conception rates.” Also, in studies designed to improve menstrual regularity, GLP-1 RAs, specifically exenatide and liraglutide, improved ovulation rate and menstrual frequency. Also, they have been shown to have anti-inflammatory effects in endometrium that is affected by PCOS, diabetes, or obesity [32]. Thus, GLP-1 RAs have been preliminarily shown to improve PCOS symptoms.

One randomized study compared whether using only T2DM medication metformin and GLP-1 RA or a mixture with current PCOS medication cyproterone acetate and ethinylestradiol (CPA/EE) would show any significant improvement in PCOS symptoms. They recruited 60 overweight or obese females with PCOS and randomized them into either a 1:1 ratio of CPA/EE + metformin or GLP-1 RA + metformin. After taking the medication for 12 weeks, the study found that both improved reproductive functions. They found that 76.66% of patients in the CPA/EE + metformin group and 73.3% of patients in the GLP-1 RA + metformin group had recovered their menstrual cycle, and both showed a statistically significant decrease in polycystic ovaries and amenorrhea (p < 0.05). Other findings for the CPA/EE + metformin group found a statistically significant decrease in multiple hormones like LH/FSH ratio, testosterone, and sex-hormone binding globulin. In terms of physical weight parameters, only the GLP-1 RA + metformin group saw statistically significant changes in BMI, weight, and waist size. This suggests that addressing both T2DM and PCOS together can be important in recovering some reproductive function [29]. It also shows that a combined treatment specifically with metformin and GLP-1 RA can be effective in alleviating some of the symptoms of both PCOS and obesity, as also discovered in a different study [33]. However, the current use of metformin may still provide better benefits than the use of GLP-1 RAs when hoping to alleviate PCOS symptoms.

Another randomized study analyzed the use of GLP-1 RA medication liraglutide in females with PCOS undergoing in vitro fertilization (IVF) treatment. The 28 females who were all infertile, obese, and had PCOS were separated into either metformin or metformin + liraglutide over a span of 12 weeks. Similarly to the previous study, both groups saw a significant change in body mass, BMI (both with p = 0.001), waist circumference (p = 0.001 and p < 0.001), and total body fat (both with p = 0.001). Patients had a decrease in body mass of 7.5 ± 3.9 kg, BMI dropped 2.7 ± 1.3 kg/m^2^, waist circumference had a noticeable decrease of 11.7 ± 9.0 cm, and total body fat loss fell below 160 cm^2^. Afterward, the investigators continued the IVF protocol to retrieve oocytes, begin the fertilization process, culture and transfer them back into the patients. Reproductive parameters were also taken and found to have statistically significant pregnancy rates per embryo transfer (p = 0.03). Specifically, the study found that two of the seven patients on metformin had successful pregnancy rates per embryo transfer compared to six out of seven patients on metformin + liraglutide [34]. This suggests that the use of GLP-1 RAs with metformin is more successful in increasing pregnancy rates per embryo transfer when compared to metformin alone in females with PCOS and a history of infertility. A similar trial investigated the natural pregnancy rate in overweight women with PCOS, using exenatide or metformin, and found that the rate was higher in those treated with exenatide [31].

Effects of GLP-1 RAs on Male Testosterone and Fertility

In individuals AMAB, a connection also exists between testosterone levels and insulin where obese men or those with T2DM have lower testosterone levels [35]. This negatively impacts male fertility due to factors such as decreased sperm count and hormonal imbalances. Not only does obesity in males impact fertility, it also is correlated with decreased success when patients attempt fertility treatment, such as IVF [36]. Thus, the weight loss effects of GLP-1 RA medications can greatly help males suffering from fertility problems.

Insulin resistance and T2DM are strongly intertwined with obesity. In turn, obesity negatively impacts testosterone and male fertility by causing secondary hypogonadism seen in Figure 3. Specifically, “excess adipose tissue leads to increased peripheral aromatization of testosterone” which then causes more estradiol production, which inhibits the HPG axis, affecting testosterone and sperm production [36]. The dysregulation of hormones such as LH, FSH, and thus testosterone leads to oligospermia and azoospermia, which contributes to male infertility [36].

One study explored whether a relationship between testosterone and insulin levels existed by observing 21 men who had neither T2DM nor reproductive disorders. To provide baseline levels, the study took an initial oral glucose tolerance test and insulin sensitivity test as well as LH sampling over a 12-hour interval. After, a GnRH antagonist was given to induce hypogonadism where the participants’ levels of LH were measured in 15-minute increments for an hour. Testosterone levels were stimulated using GnRH and human chorionic gonadotropin (hCG). In this second phase, testosterone levels were measured at the 24- and 48-hour marks and found to have increased from 115 to 677 ng/dl and 74 to 1230 ng/dl. During the stimulation of testosterone, the study took fasting glucose and found that nine remained within normal limits, nine had impaired tolerance, and three had T2DM. This suggests a strong correlation between insulin sensitivity and Leydig cell function given that an increase in both testosterone and insulin remained even 48 hours after GnRH and hCG administration. Overall, this indicates that insulin resistance may be associated with decreased Leydig cell testosterone secretion in men due to its role in promoting GnRH secretion and stimulating LH and FSH stimulation [35]. Although the relationship between insulin and testosterone can enlighten the pathophysiology involved in male infertility, this study used a small sample size of 18 which limits its statistical power.

Another study further specifies the insulin-testosterone relationship by evaluating the effects of GLP-1 RAs on Leydig cells from 18 rats. Testosterone has an important role in promoting spermatogenesis and sperm maturation; therefore, a decrease in levels can result in poor fertility. All Leydig cells were eliminated and then rested for 14 days followed by separation into three groups: control with normal saline, 10, or 100 doses of GLP-1 RA over a span of 14 days. The study found a statistically significant increase in testosterone levels in both GLP-1 RA groups (p < 0.05) with an upregulated expression of genes such as Scarb1, Cyp11a1, and Hsd11b1 which are specific to Leydig cell maturation and steroidogenesis [37]. Although conducted on rats, it could help to elucidate a potential biomechanism between testosterone, Leydig cell maturation, and GLP-1 RA. While this study provides insight into the potential impact of male infertility, it is important to evaluate the results in the setting of human Leydig cells or the potential impact during fertility treatments.

GLP-1 RAs are also important as GLP-1 receptors are present in Sertoli and Leydig cells. A study by Rago et al. showed that sperm is a specific GLP-1 target, and that GLP-1 RAs can mediate sperm motility [36]. This shows promise in GLP-1 RAs, specifically in combating infertility. Also, current research suggests that GLP-1 RA may have stimulatory effects on the HPG axis and can combat hypogonadism that results from conditions such as obesity or PCOS. However, more research on GLP-1 receptor locations in the male reproductive system is needed [32]. The findings are summarized in Table 3.

**Table 3:** Benefits and risks on the female and male reproductive system B: Benefits; R: Risks; HbA1c: Hemoglobin A1c; LDL: Low-density lipoproteins; LH: Luteinizing hormone; BMI: Body mass index; AST: Aspartate aminotransferase; OGTT: Oral glucose tolerance test; SHBG: Sex hormone binding globulin; IVF: In Vitro Fertilization; B-hCG: Beta-human chorionic gonadotropin hormone; GLP-1 RA: Glucagon-like peptide –1 receptor agonist; DPP4: Dipeptidyl peptidase 4; PCOS: Polycystic ovarian syndrome; USA; United States of America; UAE; United Arab Emirates.

Author & year	Study population	Country	Benefits/Risks
Liraglutide: Pugliese et al., 2023 [27]	Review	Italy	B: Reduces visceral adipose tissue, waist circumference, fasting plasma glucose, HbA1c, and LDL cholesterol.
Orlistat, Exenatide, Dulaglutide, Liraglutide: Pavli et al., 2024 [28]	Review	Greece	B: Orlistat: better hormonal profile, ovulation, conception, sperm count, and motility. Dulaglutide: better ovarian function in rats with PCOS Exenatide & Liraglutide: more weight loss, improvement in female fertility
Liraglutide + metformin combination treatment: Liao et al., 2024 [29]	N = 60	China	B: Improves menstrual cycles, decreasing polycystic ovaries, and amenorrhea by upregulation of protein expression and oxidative detoxification. Decreased LH, BMI, weight, waist size, and AST. R: GI side-effects. Downregulated response to reactive oxygen species and variable platelet degranulation.
Liraglutide, exenatide: Cena et al., 2020 [31]	N = 446	Italy	B: Weight loss = higher retrieved eggs for IVF. Increase in pregnancy rate, greater effects on menstrual frequency, total testosterone, SHBG, insulin versus metformin. R: Weight loss not correlated to positive B-hCG, pregnancy.
liraglutide, sitagliptin, etc.: Jensterle et al., 2019 [32]	Review	Slovenia & Netherlands	B: anti-inflammatory and anti-fibrotic effects on gonads and endometrium lead to improved menstruation & fertility rates.
Liraglutide: Bader et al., 2024 [33]	N = 486	UAE	B: Combination therapy (liraglutide + metformin): more effective than monotherapy in weight loss treatment. GLP-1 RA: greater effects on endocrine parameters (menstrual frequency, pregnancy rate) than metformin
Liraglutide: Salamun et al., 2018 [34]	N = 28	Slovenia	Decreased BMI, waist circumference, visceral adipose tissue, fasting and post-OGTT, and increased SHBG. Improved insulin sensitivity leads to balanced secretion of LH and ovarian androgens restoring some ovarian and endometrial function. A 69.2% of previously infertile patients were pregnant within 1 year either spontaneously or IVF procedures
Liraglutide and Tirzepatide: Varnum et al., 2023 [36]	SCALE trial: N = 846 pts SURMOUNT trial: N = 2539	Italy & USA	SCALE Benefits: use of liraglutide vs placebo - weight loss over 46 weeks. SURMOUNT benefits: tirzepatide - weight loss over 72 weeks. SCALE risks: Need more studies to evaluate efficacy SURMOUNT risks: Gastrointestinal adverse effects
Li et al., 2022 [37]	N = 21	China	B: Increased testosterone levels and Leydig cell maturation. R: Many genes downregulated, and impacts still unclear

Effects of GLP-1 RAs on the neurological system

Throughout many conducted literature reviews, it has been found that there are numerous anti-inflammatory properties associated with the GLP-1 receptor present in the brain. Studies included reports that focused on multiple diseases related to aging and mechanisms including, GLP-1 once bound to the G protein-coupled receptor, leading to the formation of memory, neuroprotection, synapse repair, and the regeneration of neurotransmitters after release [38]. Though continuous research is being conducted on its movement across the blood-brain barrier through endothelial cells, tanycytes, and the choroid plexus, there is evidence that activation of these pathways leads to autophagy, synapse formation, and the reduction of inflammatory responses [39].

The use of liraglutide, a GLP-1 agonist, in a recent randomized, placebo-controlled clinical trial with twenty metformin-treated obese prediabetic or type 2 newly diagnosed diabetic patients, included in a literature review by Wilbon et al. in 2023, showed that patients treated with liraglutide presented with an increase in memory scores after weight loss and glycemic control comparisons (mean memory z-score from -0.67 to 0.032, p = 0.0065) [40]. Additionally discussed in the same literature review, a separate preclinical trial with two-month-old amyloid precursor protein/presenilin 1 (APP/PS1) mice showed that liraglutide increased the number of synapses and memory retention [40]. Another study analyzing the impact on Parkinson's disease in cellular and animal models discovered that the GLP-1 receptors have neuroprotective effects on the dopamine pathways as the drug is able to increase phosphorylated adenosine monophosphate-activated protein kinase (p-AMPK) expressions and also reduce the nuclear factor-kappa B (NF-kB) protein levels [38]. In an alternative source discussed in the same literature review by Diz-Chaves et al., dulaglutide administered prophylactically or semi-therapeutically reduced lymphocytic infiltration into the CNS in experimental autoimmune encephalomyelitis (EAE) mice in a study of multiple sclerosis with p<0.01 for prophylactic treatment and p<0.0001 for semi-therapeutic medication [41].

Contributing to the new research developments, liraglutide also has shown positive results in decreasing active microglia count in the hippocampus and cortex and the amyloid plaque proximity numbers in APP/PS1xdb/db and APPswe/PS1dE9 mice [38]. In addition to existing clinical trials examining the effects of GLP-1 on aging-related diseases of the brain, a rat model preclinical trial using rats on a standard diet or high-fat diet with streptozotocin injection also represented the advancing neurological progress that the agonists have on spatial learning, cognitive impairment, and memory. The results from this study revealed that rats with diabetes mellitus treated with tirzepatide performed better in spatial learning and memory impairment than the group without medications during an escape platform and previous experiment memory retention trial [42]. Amyloid beta protein accumulation in the hippocampus was also significantly reduced in rats with diabetes treated with tirzepatide with p values at least <0.01 [42].

Parab et al. examined the efficacy and safety of sodium/glucose cotransporter-2 inhibitors (SGLT2i), GLP-1 RA, and dipeptidyl peptidase 4 inhibitors (DPP4i). It was discovered that just GLP-1 RAs were connected to a lower chance of stroke in comparison with placebo (RR: 0.85, 95% CI: 0.76-0.94) [43].

With extensive reviews focusing on GLP-1 RA and its effects on the brain, it has also been found that there have been instances of psychiatric adverse events (AEs) with its use. Based on cases from Q1 2004 and Q1 2023 in the FDA Adverse Event Reporting System (FAERS) database, with a higher instance occurring in women (65.89%) vs. men (30.96%), a pharmacovigilance study found 8,240 GLP-1 RAs-associated psychiatric AEs out of 181,238 AE reports [42-44]. Out of the 8,240 psychiatric AEs, 47.91% were due to exenatide, 22.25% due to dulaglutide, 13.98% due to liraglutide, 12.54% due to semaglutide, 3.18% due to tirzepatide, and 0.15% were due to lixisenatide [44]. The study analyzed the psychiatric AEs using reporting odds ratio (ROR) through disproportionality analysis and eight different AEs were categorized as follows: self-induced vomiting (ROR = 3.77, 95% CI = 1.77-8.03), fear of eating (ROR 3.35, 95% CI = 1.65-6.78), binge eating (ROR = 2.70, 95% CI = 1.75-4.16), sleep disorder due to general medical condition-insomnia type (ROR = 2.01, 95% CI = 1.60-2.52), nervousness (ROR = 1.97, 95% CI = 1.85-2.11), fear of injection (ROR = 1.96, 95% CI = 1.60-2.40), stress (ROR = 1.28, 95% CI = 1.19-1.38), and eating disorder (ROR = 1.57, 95% CI = 1.40-1.77) [42-44]. A case series of incorrect administration of semaglutide looked at three different events, with one case involving neurological effects. Instead of 0.1 mL (0.25 mg) of subcutaneous administration of semaglutide 2.5 mg/1 mL, a 37-year-old female with a history of obesity self-administered her first dose of 1 mL (2.5 mg) [45]. After frequent vomiting that was resolved in 24 hours, the patient experienced constant headache, diminished appetite, weakness, and fatigue for the following three days, but a lack of patient follow-up after four days limits the indication of symptom duration [45]. There are concerns regarding increased depression and potential suicidality with these medications, which are currently being investigated by the FDA [46].

In a meta-analysis of 233 clinical trials published between 2004 and 2017 looking at 147,710 type 2 diabetic patients and nine treatments - two incretin-based therapies, one placebo, and six traditional antidiabetic drugs (metformin, insulin, sulfonylurea, thiazolidinediones, alpha-glucosidase inhibitor, and sodium-glucose co-transporter 2) - GLP-1 RA use increased dizziness risk when compared to insulin and placebo with an odds ratio of 2.06 and 1.39 [47]. GLP-1 RAs also increased the risk of headaches when compared to insulin, thiazolidinediones, and placebo with the odds ratio of 1.18 to 1.41 [47]. Among the nine diabetic treatments, GLP-1 RAs were ranked lowest in safety regarding dizziness (ranked 8th) and headache (ranked 9th) [47]. One study of 9,901 patients with a new diagnosis of T2DM observed a non-significant reduction in cognitive impairment (p=0.11) with the dulaglutide sc. vs. placebo in Cukierman-Yaffe et al., but with posthoc adjustments for standardized baseline scores of individuals, there was a further reduction in cognitive outcomes by 14% (p=0.0018) [48]. The findings are summarized in Table 4.

**Table 4:** Benefits and risks on the neurological system B: Benefits; R: Risks; MS: Multiple sclerosis; EAE: Experimental autoimmune encephalomyelitis; CNS: Central nervous system; APP/PS1: Amyloid precursor protein/presenilin 1; GLP-1 RA: Glucagon-like peptide –1 receptor agonist; AEs: adverse events; FAERS: Food and Drug Administration Adverse Event Reporting System; ROR: reporting odds ratio; CI: confidence interval

Author & year	Study population	Country	Benefits/Risks
Dulaglutide: Diz-Chaves et al., 2024 [38]	Preclinical MS EAE mice trial (prophylactic vs semi-therapeutic medication)	Spain	B: Suppresses development of Th1/Th17 cells in the CNS; reduced lymphocytic infiltration into the CNS & modulates T cell pathogenicity
Liraglutide: Wilbon et al., 2023 [40]	N = 20	USA	B: Neuroprotective properties in the CNS through the blood-brain barrier with discrimination of direct drug effect and positive weight loss; increased memory scores
Tirzepatide: Guo et al., 2023 [42]	Preclinical high-fat diet and streptozotocin injected diabetic rat trial (standard diet vs high-fat diet)	China	B: Hippocampal synaptophysin protein synthesis + neuronal protection + increased density of dendritic spines; spatial learning and memory impairment improvement; inhibition of amyloid beta protein accumulation
Exenatide, dulaglutide, liraglutide, etc.: Chen et al., 2024 [44]	N = 8,240	China	R: Mechanism unclear; Self-induced vomiting, fear of eating, binge eating, sleep disorder due to a general medical condition–insomnia type, nervousness, fear of injection, stress, and eating disorder
Exenatide, Liraglutide, Albiglutide, Lixisenatide, Dulaglutide: Gao et al., 2019 [47]	N = 147,710	China	R: Postprandial use may increase blood flow in cerebral regions and may reduce blood pressure; increased dizziness risk when compared to insulin and placebo (odds ratio of 2.06 and 1.39); increased risk of headaches when compared to insulin, thiazolidinediones, and placebo (odds ratio of 1.18 to 1.41)
Dulaglutide: Cukierman-Yaffe et al., 2020 [48]	N = 9,901	24 countries	R: Cognitive impairment by 14% (p=0.0018) after post-hoc adjustments

Effects of GLP-1 RAs on the gastrointestinal system

GLP-1 RA has been shown to be effective in improving glycemic control; hence, they are in use presently for treating T2DM. The treatment is normally accompanied by a set of gastrointestinal side effects. Nausea and diarrhea are observed as common adverse effects found in 1 in 10 patients [49]. Other adverse effects include vomiting, constipation, abdominal pain, and dyspepsia in patients from 1-10 out of 100. Nearly 50% of patients receiving GLP-1 RA reported nausea as the most common side effect [49].

Consistent with the evidence available in the literature, in this large-scale real-world study, it is observed that there is a more than twofold increased risk of gastrointestinal adverse events associated with GLP-1 RA. These included the most commonly reported AEs in FAERS - nausea (8,988; 42.23%), diarrhea (4,666; 21.93%), and vomiting (4,660; 21.90%). The results were consistent with a systematic review in which researchers identified gastrointestinal adverse drug reactions (ADRs) as the main adverse effects of GLP-1 RAs, with nausea and diarrhea having prevalence rates of up to 51% and 20% of patients, respectively and these were observed to be highly specific to three GLP-1 RAs: liraglutide, dulaglutide, and semaglutide [50].

Most of these AEs are presumed to result from a drug's action in mechanisms involving delayed gastric emptying and enhanced neural pathways [50]. The prevalence of these gastrointestinal symptoms makes the proactive response and management of these symptoms very critical toward fostering patient compliance and treatment outcomes.

A meta-analysis that included 25 studies related to GLP-1 RA, specifically exenatide and liraglutide, showed no significant risk for acute pancreatitis in patients with T2DM [51]. The odds ratio for acute pancreatitis was, in the case of exenatide, 0.84 (95% CI 0.58-1.22), and for liraglutide, it was 0.97 (95% CI 0-21-4.39), both not showing any significant increase in risk. The study authors also stated that there was no adequate evidence at the time to conclude that there was an increased risk of acute pancreatitis due to GLP-1 RA, although they emphasized the importance of monitoring rare, long-term adverse events in future studies [51].

GLP-1 RA use did not increase the risk of pancreatitis when compared to control groups, according to a systematic review of 55 randomized controlled trials with a sample size of 33,350 [52]. Three retrospective cohort studies that observed 1,466 cases of pancreatitis and found no evidence of an acute pancreatitis risk in the context of GLP-1 RA corroborated these findings [53-55]. Therefore, the strongest support for the presumed pancreatic safety of GLP-1 RAs in clinical practice comes from a combination of observational studies and randomized trials [52].

Semaglutide, a GLP-1 RA, has shown remarkable efficacy in treating T2DM, obesity, and related conditions, but its impact on the gastrointestinal (GI) system is a notable concern. GI side effects, including nausea, vomiting, diarrhea, and constipation, are among the most commonly reported adverse events. These effects are dose-dependent and most pronounced in the initial stages of treatment [56]. Tirzepatide significantly improved the clamp disposition index from 0.3 to 2.3 pmol m⁻² L min⁻² kg⁻¹ (ETD vs placebo: 1.92 [95% CI 1.59-2.24]; p < 0.0001) and outperformed semaglutide (ETD: 0.84 [95% CI 0.46-1.21]). It also showed greater improvements in insulin secretion rate (ETD: 102.09 pmol min⁻¹ m⁻² [95% CI 51.84-152.33]) and insulin sensitivity (ETD: 1.52 mg min⁻¹ kg⁻¹ [95% CI 0.53-2.52]) compared to semaglutide [56]. On meal tolerance testing, tirzepatide reduced glucose excursions more effectively than semaglutide and placebo. GI adverse events were common across groups, with nausea (24%, 30%, and 25%), diarrhea (20%, 30%, and 21%), and vomiting (7%, 11%, and 4%) reported for tirzepatide, semaglutide, and placebo, respectively [56].

Gradual dose escalation can mitigate their severity, yet approximately 10% of patients discontinue treatment due to intolerable symptoms. Semaglutide commonly causes GI side effects such as nausea, vomiting, and diarrhea, with higher doses increasing the frequency of these events. In subcutaneous formulations, nausea affected 11.4%-20% of patients, vomiting 4%-11.5%, and diarrhea 4.5%-11.3%, compared to significantly lower rates in placebo groups (p < 0.05). Oral formulations showed similar trends, with GI disturbances occurring in up to 23.2% of patients depending on the dose. Interestingly, these GI disturbances may also play a role in semaglutide's weight loss effects by reducing appetite and food intake, suggesting a dual mechanism of action that blends efficacy with tolerability challenges [57].

Higher doses of semaglutide, such as the 0.4 mg dose evaluated in the treatment of non-alcoholic steatohepatitis (NASH), have been associated with increased GI disturbances. For instance, a 72-week trial demonstrated that while the 0.4 mg dose resolved NASH in 59% of patients, the frequency and severity of nausea, constipation, and vomiting were significantly greater compared to lower doses and placebo [58]. This demonstrates the trade-off between achieving therapeutic efficacy and maintaining patient adherence due to tolerability concerns. Additionally, semaglutide has been linked to an increased risk of gallbladder-related diseases, particularly at higher doses, further complicating its GI safety profile [58]. In Newsome et al.’s study, 320 patients given semaglutide at a dose of 0.4 mg achieved NASH resolution with no worsening of fibrosis in 59% of patients, compared to 17% in the placebo group (P<0.001). The 0.4-mg group also showed a 43% improvement in fibrosis, though this difference from placebo (33%) was not statistically significant (P = 0.48); the mean weight loss in the 0.4-mg group was 13%, compared to 1% in the placebo group [58]. However, the 0.4-mg dose was associated with higher rates of nausea, constipation, and vomiting, and 1% of patients receiving semaglutide developed malignant neoplasms.

Despite these challenges, semaglutide’s GI effects are not unique among GLP-1 RA. Comparative studies indicate that semaglutide may have a slightly better safety profile than other agents in this class, such as liraglutide and exenatide, which are associated with stronger signals for adverse outcomes, including pancreatic carcinoma. However, semaglutide is not without risks; ongoing research is needed to clarify its potential long-term implications, including its role in pancreatic health; a total of 3073 pancreatic carcinoma cases were associated with GLP-1 RA, with liraglutide showing the strongest signal for pancreatic cancer (ROR 54.45, PRR 52.52) [59]. Exenatide and lixisenatide also had stronger signals than semaglutide and dulaglutide, with exenatide showing the highest mortality rate (63.6%). The potential mechanisms behind GLP-1 RA-related pancreatic carcinoma include cAMP/protein kinase activation, Ca2+ channel modulation, endoplasmic reticulum stress, and oxidative stress [59]. These findings suggest varying degrees of risk for pancreatic carcinoma associated with different GLP-1 RAs.

Overall, while semaglutide's GI effects limit its tolerability, they also appear to contribute to its efficacy, particularly in weight management. Balancing these effects is crucial, especially in clinical scenarios requiring higher doses for conditions like NASH or advanced metabolic dysfunction [60]. Optimizing dosing strategies, closely monitoring patients, and tailoring treatment to individual needs are essential to maximizing semaglutide's benefits while minimizing its drawbacks. The findings are summarized in Table 5.

**Table 5:** Benefits and risks on the gastrointestinal system B: Benefits; R: Risks; GLP-1 RA: Glucagon-like peptide-1 receptor agonists; GI: Gastrointestinal

Author & year	Study population	Country	Benefits/Risks
GLP-1 RAs: Liu et al., 2022 [50]	N = 28,314	China	B: Improved glycemic outcomes. R: Nausea (42.23%), diarrhea (21.93%), vomiting (21.90%) caused by delayed gastric emptying and neural pathway effects.
Exenatide, liraglutide: Alves et al., 2012 [51]	N = 776,980	Portugal	B: Glycemic control without increased acute pancreatitis risk.
Semaglutide: Heise et al., 2022 [56]	N = 117	Germany	R: Nausea, vomiting, diarrhea, constipation, dose-dependent, most pronounced in early treatment.
Semaglutide: Newsome et al., 2021 [58]	N = 320	England	R: nausea, vomiting, constipation; potential risk of gallbladder disease; malignancy in 1% of patients.
Semaglutide: Cao et al., 2023 [59]	N = 3073	China	R: Pancreatic carcinoma risks: Exenatide shows the highest mortality (63.6%). Potential cAMP, Ca2+ channel, and oxidative stress mechanisms.
Semaglutide: Bandyopadhyay et al., 2023 [60]	N = 2413	India	B: Improve liver enzymes, and metabolic parameters for NASH/NAFLD patients and reduce liver stiffness

Effects of GLP-1 RAs on the cardiovascular system

Cardiovascular diseases are a leading cause of mortality and morbidity worldwide. The close link between diabetes and cardiovascular disease has become a critical focus in evaluating the potential of glucagon receptor antagonists to reduce cardiovascular events. This connection highlights the importance of targeting metabolic pathways to improve cardiovascular outcomes in high-risk populations [61]. GLP-1 RAs have emerged as a significant treatment option for cardiovascular diseases. Several studies have shown the impact of GLP-1 RAs in reducing major adverse cardiovascular events (MACE) [62].

GLP-1 RAs in cardiovascular disease can be related to several mechanisms. Maintaining stable blood glucose levels can mitigate the adverse effects of hyperglycemia on blood vessels and contribute to cardiovascular disease (CVD) risk reduction in diabetic patients. In addition, new evidence proposes that GLP-1 receptors are not confined to pancreatic islets but are also expressed in cardiomyocytes and vascular endothelial cells and promote myocardial glucose uptake and utilization, reduce oxidative stress, and inhibit cardiomyocyte apoptosis. Another important role of the GLP-1 receptor activation is to induce vasodilation through several pathways. It stimulates the production of endothelial nitric oxide, a potent vasodilator, and may modulate the renin-angiotensin-aldosterone system (RAAS) for further influencing vascular tone and blood pressure regulation. Many studies have proven that GLP-1 RAs consistently lead to weight loss, they also have an anti-inflammatory effect, which significantly reduces inflammatory biomarkers [61]. Ongoing analyses focus on identifying patient subgroups with varying risk profiles to determine which populations derive the greatest benefit from GLP-1 RA therapy [62].

A meta-analysis of 89,790 T2DM in patients with pre-existing CVD demonstrated a 14% reduction of MACE (HR: 0.86; CI: 0.80-0.93) on semaglutide, while those at high risk of CVD, but without a history of cardiovascular events, seemed to have minimal or no effect (HR: 0.94; CI: 0.82-1.07). This study discovered that the baseline HbA1c level was an effective modifier. Participants with baseline HbA1c levels that were equal to or higher than 8% showed considerable decreases in the risk of MACE compared with other groups [62]. In another study, a total of 3297 patients were randomized, 83% of patients had established cardiovascular disease with a mean HgA1c of 8.7%. There was a slight reduction in the cardiovascular outcomes relative to the placebo group. Furthermore, there were fewer non-fatal strokes in the semaglutide group (1.6%) relative to the placebo group (2.7%), demonstrating its efficacy in reducing unfavorable cardiovascular outcomes. Some GLP RAs such as Liraglutide have also significantly reduced the risk of nephropathy and retinopathy events. The study demonstrated that new or worsening nephropathy occurred in 3.8% of patients in the semaglutide group relative to 6% in the placebo group [63].

Obesity is an important factor in cardiovascular events. GLP-1 RAs have demonstrated a vital role in addressing cardiovascular end outcomes among overweight and obese patient populations. In a randomized, placebo-controlled study assessing cardiovascular end outcomes in patients 45 years and older with preexisting cardiovascular conditions and BMI greater than or equal to 27 but no history of diabetes, there were fewer cardiovascular endpoints in patients on semaglutide (6.5%) relative to placebo (8.0%) (0.72 to 0.9, 95% confidence interval; P<0.001) [64].

In a randomly assigned study, 529 patients with heart failure (HF) with preserved ejection fraction and a BMI of 30 or higher were randomly assigned to receive once weekly semaglutide (2.4 mg) or placebo for 52 weeks. The change in body weight and Kansas City Cardiomyopathy Questionnaire Clinical Summary Score (KCCQ-CSS) scores range from 0 to 100, with higher scores indicating fewer symptoms and physical limitations were used to assess the outcome. The result showed that the mean change in the KCCQ-CSS was 16.6 points with semaglutide and 8.7 points with placebo (estimated difference, 7.8 points; 95% confidence interval [CI], 4.8 to 10.9; P<0.001), and the mean percentage change in body weight was −13.3% with semaglutide and −2.6% with placebo (estimated difference, −10.7 percentage points; 95% CI, −11.9 to −9.4; P<0.001). The mean change in the 6-minute walk distance was 21.5 m with semaglutide and 1.2 m with placebo (estimated difference, 20.3 m; 95% CI, 8.6 to 32.1; P<0.001). In the analysis of the hierarchical composite endpoint, semaglutide produced more wins than placebo (win ratio, 1.72; 95% CI, 1.37 to 2.15; P<0.001). The mean percentage change in the CRP level was -43.5% with semaglutide and -7.3% with placebo (estimated treatment ratio, 0.61; 95% CI, 0.51 to 0.72; P<0.001). Serious adverse events were reported in 35 participants (13.3%) in the semaglutide group and 71 (26.7%) in the placebo group [65]. In another study, 300 patients with HF and reduced ejection fraction who were recently hospitalized for HF with and without diabetes were randomized to liraglutide or placebo. After 180 days, there was no difference in death rate or HF hospitalization [66].

Lowering blood pressure is an important factor in reducing cardiovascular events. GLP-1 RA leads to a reduction in systolic BP of 2 to 6 mm Hg, and blood. Meta-analyses have shown that a systolic blood pressure reduction of 10 mm Hg was associated with approximately 20% reduction of MACE [66].

In addition, various experimental models of atherosclerosis have shown that GLP-1 RA reduces the development and progression of atherosclerotic lesions by its antiatherogenic and anti-inflammatory effects on endothelial cells, monocytes, and macrophages, as well as vascular smooth muscle cells. The anti-inflammatory properties of GLP-1 RAs were shown by clinical data in a small population of patients with liraglutide. Results show decreased production of TNF-α and interleukin-1 in isolated human peripheral blood mononuclear cells. GLP-1 RA has been shown to reduce systemic inflammation as measured by levels of C-reactive protein [66].

Various studies have highlighted the significant benefits of GLP-1 RAs in reducing cardiovascular disease. While further research is needed to fully explore their advantages and assess their effects across diverse populations, the evidence shows the importance of considering the therapeutic advantages of GLP-1 RAs for specific patients. It is vital that we determine the primary target population for GLP-1 RAs. The findings are summarized in Table 6.

**Table 6:** Benefits and risks on the cardiovascular system B: Benefits; R: Risks; GLP-1 RA: Glucagon-like peptide-1 receptor agonist; CVD: Cardiovascular disease; HF: Heart failure; HHF: Heart failure hospitalization; CVOTs: Cardiovascular outcome trials; MACE: Major Adverse Cardiovascular Events; KCCQ-CSS: Kansas City Cardiomyopathy Questionnaire Clinical Summary Score

Author & year	Study population	Country	Benefits/Risks
Semaglutide, liraglutide: Ferhatbegović et al., 2023 [61]	N = 60080	Bosnia and Herzegovina	B: Anti-inflammatory effects, weight loss, blood pressure, and blood lipid control
Semaglutide & Liraglutide: Sheahan et al., 2020 [63]	N = 3232	United States	B: Reduction in risk of nephropathy and retinopathy events as well as reducing cardiovascular end outcomes
Semaglutide: Lincoff et al., 2023 [64]	N = 17604	Denmark, United Kingdom, United States	B: Reduction in cardiovascular outcomes for patients of BMI> 27 without T2DM
Semaglutide: Kosiborod et al., 2023 [65]	N = 529	United Kingdom,	B: Anti-inflammatory effects relative to placebo
Liraglutide: Marx et al., 2022 [66]	Review	Canada, Germany, United Kingdom	B: Reduce systemic inflammation, reduction of 10 mmHg in systolic blood pressure.

## Conclusions

GLP-1 RAs exhibit significant effects across various organ systems, extending their therapeutic utility beyond traditional glycemic control, particularly in dermatological, endocrine, reproductive, neurological, gastrointestinal, and cardiological contexts. In the integumentary system, GLP-1 RAs show promise in managing psoriasis and enhancing diabetic wound healing through anti-inflammatory pathways and keratinocyte migration, but they can cause rare dermatological side effects like hypersensitivity reactions and eosinophilic panniculitis. GLP-1 RAs reduce pancreatic cancer risk within the endocrine system and offer nephroprotective benefits. However, they also present a debated risk of medullary and papillary thyroid cancer, especially with long-term use. This finding stresses the need for vigilant monitoring and further research to refine their safety profiles and uncover other potential adverse effects. GLP-1 RAs improve fertility outcomes for the reproductive system by alleviating PCOS symptoms and enhancing ovulation in females while increasing Leydig cell function in males. However, more research is needed to generalize these findings. Neurologically, GLP-1 RAs demonstrate neuroprotective effects, reducing neuroinflammation and improving cognitive function in conditions like Alzheimer's and Parkinson's disease, but their association with dizziness, headaches, and psychiatric adverse effects underscores the importance of patient-specific evaluations. In the gastrointestinal system, GLP-1 RAs effectively manage glucose levels and support weight loss, but their dose-dependent side effects, such as nausea, vomiting, and diarrhea, can significantly impact treatment adherence. While prior investigations suggested an association between GLP-1RA use and pancreatic carcinoma, our analysis found no increased risk in the endocrine pancreas. Instead, sporadic cases were observed in the exocrine pancreas, suggesting that future studies should distinguish between these divergent outcomes. Finally, cardiological studies reveal their ability to reduce MACE through vasodilatory, anti-inflammatory, and anti-atherogenic effects, with specific efficacy in managing heart failure and reducing blood pressure. These findings depict the versatile therapeutic potential of GLP-1 RAs while calling for the importance of personalized treatment strategies and long-term safety monitoring across diverse patient populations.
